# Аналитический обзор результатов мониторинга основных эпидемиологических характеристик йододефицитных заболеваний у населения Российской Федерации за период 2009–2018 гг.

**DOI:** 10.14341/probl12433

**Published:** 2021-04-09

**Authors:** Е. А. Трошина, Н. М. Платонова, Е. А. Панфилова

**Affiliations:** Национальный медицинский исследовательский центр эндокринологии; Национальный медицинский исследовательский центр эндокринологии; Национальный медицинский исследовательский центр эндокринологии

**Keywords:** йододефицитные заболевания, щитовидная железа, йодированная соль, профилактические мероприятия, зоб, закон

## Abstract

ОБОСНОВАНИЕ. Уровень потребления йода населением во многом определяет спектр тиреоидной патологии. На сегодняшний день в РФ йододефицитные заболевания (ЙДЗ) занимают лидирующие позиции в структуре всех заболеваний щитовидной железы (ЩЖ). Хронический дефицит йода (ЙД) приводит к неблагоприятным последствиям для здоровья и существенным экономическим затратам на их устранение в масштабах страны. Кроме того, спектр тиреоидной патологии в условиях ЙД не ограничивается проблемой ЙД, и изучение остальных заболеваний ЩЖ также представляет интерес.ЦЕЛЬ. Проанализировать динамику основных эпидемиологических показателей ЙДЗ и других заболеваний ЩЖ у всего населения РФ за период 2009–2018 гг., используя данные официальной государственной статистики.МЕТОДЫ. Проанализированы заболеваемость и распространенность заболеваний ЩЖ у всего населения РФ с использованием данных официальной государственной статистики. Использованы статистические формы №63 МЗ СР РФ «Сведения о заболеваниях, связанных с микронутриентной недостаточностью» и №12 «Сведения о числе заболеваний, зарегистрированных у больных, проживающих в районе обслуживания лечебного учреждения, РОССТАТ». Анализируемый период — 2009–2018 гг. Для оценки динамики распространенности и заболеваемости были построены линейные регрессионные модели.РЕЗУЛЬТАТЫ. Данные проанализированы в соответствии с представленной в статистической отчетности структурой заболеваний: зоб, тиреоидит, гипотиреоз, тиреотоксикоз, синдром врожденной йодной недостаточности. За десятилетний период 2009–2018 гг. отмечается статистически значимый рост распространенности различных форм зоба, тиреотоксикоза, синдрома врожденной йодной недостаточности у всего населения РФ. В течение периода наблюдения выявлен рост заболеваемости тиреотоксикозом. В отношении заболеваемости синдромом врожденной йодной недостаточности выявлена лишь тенденция к росту. Несмотря на то что в течение периода наблюдения число новых случаев различных форм зоба уменьшилось, распространенность зоба среди населения РФ остается высокой: 1,2% населения к 1 января 2019 г. В отношении тиреоидита выявлен статистически значимый рост распространенности и заболеваемости, что может быть связано с ростом аутоиммунной патологии, совершенствованием уровня диагностики, а также, в некоторых случаях, с гипердиагностикой (например, при постановке диагноза аутоиммунного тиреоидита у лиц с бессимптомным носительством антитиреоидных антител). Существующие на сегодняшний день подходы к йодной профилактике недостаточно эффективны.ЗАКЛЮЧЕНИЕ. Результаты проведенного анализа свидетельствуют преимущественно о росте распространенности тиреоидной патологии среди населения РФ на фоне проводимых региональных мероприятий. Проблема ЙДЗ остается нерешенной, что диктует необходимость внедрения всеобщего обязательного йодирования соли на территории РФ.

## Abstract (en)

BACKGROUND: The level of iodine consumption by the population largery determines the spectrum of thyroid pathology. To date, in the Russian Federation, iodine-deficiency disorders (IDD) occupy a leading position in the structure of all thyroid diseases. Chronic ID leads to adverse health consequences and significant economic costs for their elimination on a national scale. However, the spectrum of thyroid pathology is not limited to the problem of ID, and the study of other thyroid diseases is also of interest.AIMS: to assess the dynamics of prevalence and incidence of IDD and other thyroid diseases in the entire population of the RF for the period 2009–2018, using official state statistics.MATERIALS AND METHODS: the main epidemiological indicators (incidence and prevalence) of thyroid diseases in the entire population of the RF were analyzed using official state statistics. The analyzed period is 2009–2018. There was no medical intervention. To assess the dynamics of prevalence and incidence, a linear regression models were constructed, the slope of the trend line was calculated.RESULTS: the data were analyzed in accordance with the structure of diseases presented in the statistical reporting: goiter, thyroiditis, hypothyroidism, thyrotoxicosis, congenital iodine deficiency syndrome. For the ten-year period 2009–2018 there is a statistically significant increase in the prevalence of various forms of goiter, thyrotoxicosis, congenital iodine deficiency syndrome in the entire population of the Russian Federation. During the observation period, an increase in the incidence of thyrotoxicosis was revealed. With regard to the incidence of congenital iodine deficiency syndrome, only an upward trend was revealed. Despite the fact that during the observation period the number of new cases of various forms of goiter decreased, the prevalence of goiter among the population of the Russian Federation remains high: 1.2% of the population by January 1, 2019. with the growth of autoimmune pathology, improvement of the level of diagnosis, and also, in some cases, with overdiagnosis (for example, when diagnosing autoimmune thyroiditis in individuals with asymptomatic carriage of antithyroid antibodies). The current approaches to iodine prophylaxis are not effective enough.CONCLUSIONS: the results of the analysis indicate mainly an increase in the high prevalence of thyroid pathology among the population of the RF against the background of regional events. The problem of ID remains unresolved, which dictates the need to introduce universal mandatory salt iodization in the territory of the RF.

## ОБОСНОВАНИЕ

Дефицит йода (ЙД) несет многочисленные негативные последствия в отношении развития и формирования организма человека. Нарушения, вызванные ЙД, объединены под названием «йододефицитные заболевания» (ЙДЗ) и являются крайне актуальной медико-социальной проблемой [1][2].

Последствия ЙДЗ включают в себя как мертворождения, самопроизвольные аборты, возникновение врожденных аномалий у плода при тяжелом ЙД, репродуктивные нарушения, так и специфические заболевания щитовидной железы (ЩЖ), такие как гипотиреоз в районах с тяжелым ЙД и тиреотоксикоз, функциональная автономия (токсический узловой, многоузловой зоб) на территории с умеренным и легкой степени тяжести ЙД. Кроме того, хронический ЙД приводит к развитию умственной и физической отсталости детей, кретинизму, а также существенно увеличивает риск радиационно-индуцированного рака ЩЖ в случае ядерных катастроф [2][3].

В 1990 г. Всемирная встреча Организации Объединенных Наций установила цель ликвидации ЙД во всем мире. С тех пор был достигнут значительный прогресс, главным образом благодаря программам всеобщего йодирования соли. К 2013 г. приблизительно 70% всех домохозяйств в мире имели доступ к йодированной соли. В 2013 г. на основании медианы йодурии (МЙ) на уровне 100–299 мкг/л (именно этот уровень свидетельствует об адекватном потреблении йода) у детей школьного возраста было определено, что 111 стран потребляли достаточное количество йода, 30 стран оставались йододефицитными [4]. В 2019 г. в докладе Глобальной сети по йоду сообщалось, что в 23 странах зафиксировано неадекватное потребление йода [5]. В настоящее время все большее число стран придерживается принципа массовой йодной профилактики. ЙД является одним из наиболее распространенных недостатков микроэлементов, которые можно предотвратить путем осуществления программы всеобщего йодирования соли [6].

Профилактика ЙД с помощью йодирования соли была признана в мире «глобальной историей успеха» [7].

В настоящее время в РФ практически на всей территории страны выявлен ЙД различной степени тяжести, фиксируются случаи врожденной йодной недостаточности (далее в статье авторы подробно описывают, что включает в себя данный термин), связанной с недостаточным потреблением йода женщиной во время беременности [8]. С 2000 г. начали проводиться региональные программы профилактики ЙДЗ в РФ. Однако данные эпидемиологических исследований свидетельствуют о недостаточной их эффективности: более чем у половины детей показатели медианной концентрации йода в моче остаются ниже нормы — МИ 82,2 мкг/л (от 17 до 125 мкг/л), а зоб наблюдается у 5,6–38% школьников [9].

Серьезные последствия длительного некомпенсированного ЙД зарегистрированы в некоторых регионах РФ к 2016 г. В частности, в Республике Тыва (регион с тяжелым природным ЙД) отмечена высокая распространенность йододефицитных тиреопатий: 4058,3 на 100 тыс. человек, в то время как средняя распространенность йододефицитных тиреопатий на всей территории РФ на тот момент составляла 2218,7 на 100 тыс. человек. Заболеваемость ЙДЗ в Республике Тыва составила 622,4 на 100 тыс. человек, на всей территории РФ — 357,5 на 100 тыс. человек (Росстат, 2016 г.). Несколько лет назад Правительством РФ приняты основы политики здорового питания с сильным уклоном на крупномасштабное обогащение пищевых продуктов. Минздравом России активизирована законотворческая работа. Доказано, что все ЙДЗ могут быть предотвращены при адекватном потреблении йода. Тем не менее профилактические мероприятия в стране не носят постоянного и систематического характера, не охватывают все население, а способы профилактики нередко не соответствуют международным стандартам. На сегодняшний день из всех стран бывшего СССР только РФ и Украина не имеют законодательного регулирования йодной профилактики.

Данный аналитический отчет продолжает серию публикаций, выпущенных в 2018 г. По сравнению с предыдущей публикацией в представленной работе обновлены данные о распространенности тиреоидной патологии, обсуждено их значение для оценки эпидемиологической ситуации и качества проводимых профилактических мероприятий по борьбе с ЙД в Российской Федерации в 2009–2018 гг., рассмотрена их динамика за последние 10 лет, приведены примеры борьбы с ЙД в других странах.

В настоящее время во всем мире двумя основными статистическими показателями, необходимыми для оценки статуса йодной обеспеченности, являются величина медианной концентрации йода в моче и доля образцов мочи с уровнем йода менее 50 мкг/л [10]. Однако в РФ отсутствуют актуальные данные необходимого масштаба (для всей территории РФ), в связи с чем данные официальной государственной статистики, с нашей точки зрения, представляют особую ценность.

Анализ данных проведен с учетом международных определений и подходов, используемых в области обработки эпидемиологической статистической информации. В исследовании рассматриваются показатели, используемые ВОЗ для анализа распространенности ЙД и эффективности мероприятий по их контролю, дано сравнение ситуации по заболеваниям в РФ и других странах мира.

## ЦЕЛЬ

Проанализировать динамику основных эпидемиологических показателей ЙДЗ и других заболеваний ЩЖ у всего населения РФ за период 2009–2018 гг., используя данные официальной государственной статистики.

Дизайн исследования: аналитическое.

## МАТЕРИАЛЫ И МЕТОДЫ

На основании запроса в МЗ РФ получены данные официальной государственной отчетности (форма федерального статистического наблюдения №12 «Сведения о числе заболеваний, зарегистрированных у пациентов, проживающих в районе обслуживания медицинской организации» и №63 МЗ СР РФ «Сведения о заболеваниях, связанных с микронутриентной недостаточностью»), отражающие абсолютное число случаев заболеваний тиреопатиями всего и впервые выявленных, суммарно у лиц обоего пола всех возрастов, на всей территории РФ за 2009–2018 гг. Анализируемые статистические данные были получены суммарно для всей территории РФ и включали статистику как стационарной, так и амбулаторной медицинской помощи.

С использованием официальных данных Росстата о численности населения, проживающего на территории РФ в указанные годы, проведен расчет основных эпидемиологических характеристик: распространенности (отношение абсолютного числа случаев заболевания к численности населения, умноженное на 100 000 человек) и заболеваемости (отношение абсолютного числа случаев впервые выявленного заболевания к численности населения, умноженное на 100 000 человек). Численность населения соответствовала количественному значению лиц, проживающих на территории РФ в данном году, обоих полов, всех возрастов.

Эпидемиологические показатели рассчитывались для отдельных заболеваний ЩЖ. Разделение на группы проведено в соответствии с представленной в статистической отчетности структурой заболеваний ЩЖ (представлены в соответствии с международной классификацией болезней 10-го пересмотра (МКБ-10)): зоб (суммарно Е01.0-2 Эндемический зоб и Е04 Другие формы нетоксического зоба), Е06 Тиреоидит, Е02–03 Субклинический гипотиреоз вследствие йодной недостаточности и другие формы гипотиреоза, Е05 Тиреотоксикоз, Е00 Синдром врожденной йодной недостаточности. Зоб и синдром врожденной йодной недостаточности — группы заболеваний, в подавляющем большинстве случаев в основе которых лежит ЙД в питании.

В группу заболеваний, именуемую «синдромом врожденной йодной недостаточности», включены эндемические состояния, связанные с ЙД в окружающей природной среде как непосредственно, так и вследствие недостаточности йода в организме матери. Некоторые из этих состояний не могут считаться истинным гипотиреозом, а являются следствием неадекватной секреции тиреоидных гормонов у развивающегося плода; может существовать связь с природными зобогенными факторами. Из представленной группы заболеваний исключен субклинический гипотиреоз вследствие йодной недостаточности (E02). Согласно МКБ-10, синдром врожденной йодной недостаточности (Е00) включает в себя:

Е00.0 Синдром врожденной йодной недостаточности, неврологическая форма (эндемический кретинизм, неврологическая форма);

Е00.1 Синдром врожденной йодной недостаточности, микседематозная форма (эндемический кретинизм: гипотиреоидный, микседематозная форма);

Е00.2 Синдром врожденной йодной недостаточности, смешанная форма (эндемический кретинизм, смешанная форма);

Е00.9 Синдром врожденной йодной недостаточности неуточненный.

При наличии тяжелого ЙД в некоторых случаях возможно развитие субклинического гипотиреоза, а форма статистической отчетности также не позволяет отделить другие причины. Тиреоидит в большинстве случаев не является следствием йодного дефицита, но занимает важную роль в структуре тиреопатий, в связи с чем анализ динамики распространенности и заболеваемости данной патологией также представляет интерес.

В дальнейшем оценивалась динамика указанных параметров при помощи статистических методов.

Для оценки динамики распространенности и заболеваемости строились линейные регрессионные модели, рассчитывался угол наклона линии регрессии (коэффициент k; количественное выражение динамики распространенности и заболеваемости), при помощи t-критерия Стьюдента проводилась оценка статистической значимости отличия k от нуля: наличие динамики считалось статистически значимым при p<0,05.

Обработка данных проводилась с использованием программ Microsoft Excel 2013, R (version 3.2.3).

## Этическая экспертиза

Исследование одобрено Локальным этическим комитетом ФГБУ «НМИЦ эндокринологии» Министерства здравоохранения Российской Федерации. Протокол №5 от 25 марта 2020 г.: «Одобрить возможность проведения научно-исследовательской работы. Планируемая научная работа соответствует этическим стандартам добросовестной клинической практики».

## РЕЗУЛЬТАТЫ

## Раздел 1. Анализ распространенности

В период 2009–2018 гг. отмечен статистически значимый рост распространенности зоба, р<0,001 (рис. 1), и синдрома врожденной йодной недостаточности, p=3,5×10-2 (рис. 2).

**Figure:**
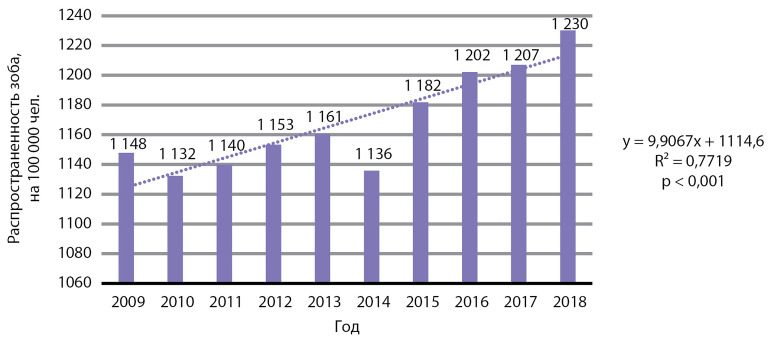
Рисунок 1.Динамика распространенности зоба, на 100 000 человек. Примечание: на данном графике и далее на всех графических изображениях представленные цифры распространенности — относительные значения, на 100 000 населения.

**Figure:**
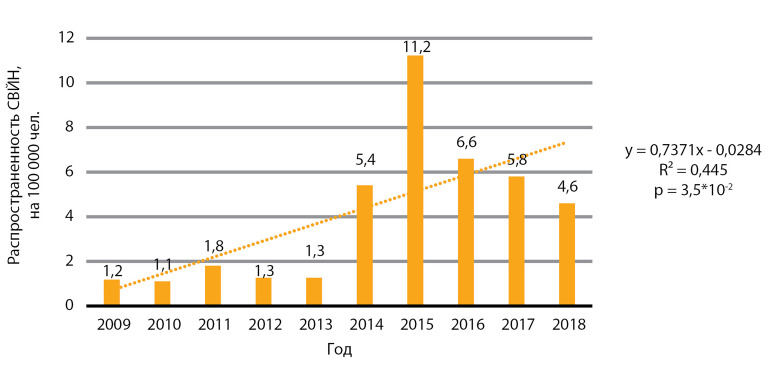
Рисунок 2. Динамика распространенности синдрома врожденной йодной недостаточности (СВЙН), на 100 000.

Данные заболевания напрямую отражают состояние проблемы ЙД в РФ. Медиана распространенности зоба за 10 лет составила 1157,0 случая на 100 000 человек, медиана ежегодного прироста распространенности — 7,5 случая на 100 000 человек. Медиана распространенности врожденной йодной недостаточности за 10 лет составила 3,2 случая на 100 000 человек, медиана ежегодного падения распространенности — 0,1 случая на 100 000 человек.

В изучаемый период также отмечен статистически значимый рост распространенности тиреоидита, р<0,001 (рис. 3). Медиана распространенности за 10 лет составила 355,6 случая на 100 000 человек, медиана ежегодного прироста распространенности — 15,9 случая на 100 000 человек.

**Figure:**
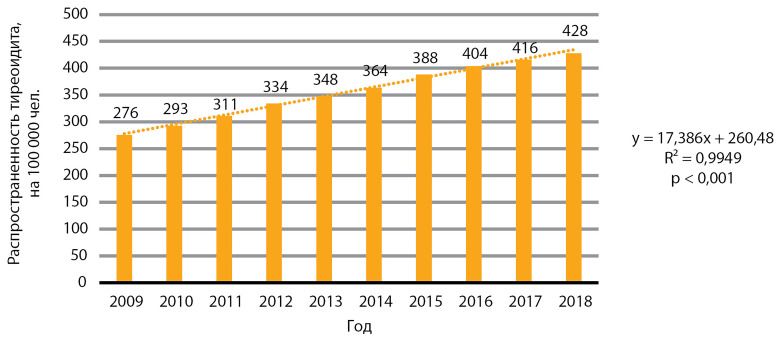
Рисунок 3. Динамика распространенности тиреоидита, на 100 000 человек.

За десятилетний период 2009–2018 гг. отмечается статистически значимый рост распространенности гипотиреоза, р<0,001 (рис. 4). Медиана распространенности за 10 лет составила 324,6 случая на 100 000 человек, медиана ежегодного прироста распространенности — 24,9 случая на 100 000 человек.

**Figure:**
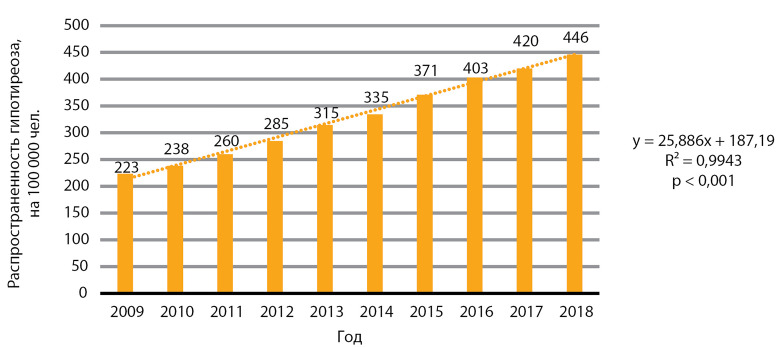
Рисунок 4. Динамика распространенности гипотиреоза, на 100 000 человек.

Согласно данным официальной отчетности, на территории РФ выявлен статистически значимый рост распространенности тиреотоксикоза, р<0,001 (рис. 5).

**Figure:**
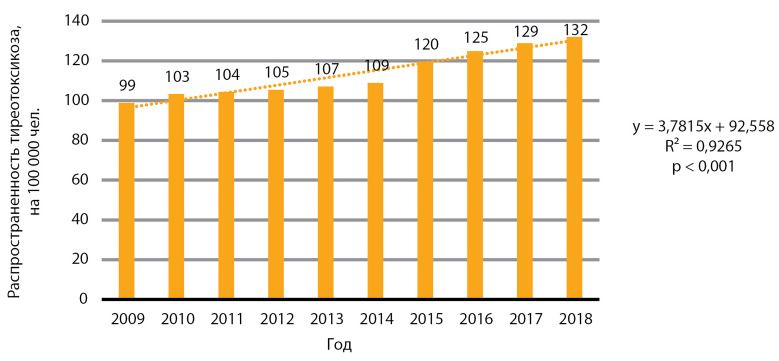
Рисунок 5. Динамика распространенности тиреотоксикоза, на 100 000 человек.

Известно, что в странах с длительным хроническим ЙД при активном внедрении программ йодной профилактики отмечается рост тиреотоксикоза вследствие декомпенсации функциональной автономии у взрослых [11]. Следует, однако, принять во внимание тот факт, что у детей и подростков тиреотоксикоз развивается преимущественно вследствие болезни Грейвса. К сожалению, имеющаяся форма статистической отчетности №12 не предполагает разделения по возрасту, что отражено в разделе «ограничения». На основании имеющихся у нас данных официальной государственной статистики и данных о численности населения (Росстат) медиана распространенности за указанный период составила 108,0 случая на 100 000 человек, медиана ежегодного прироста распространенности — 3,1 случая на 100 000 человек.

## Раздел 2. Анализ заболеваемости

На рис. 6 продемонстрирована динамика заболеваемости зобом на 100 000 человек: отмечено статистически значимое снижение заболеваемости указанной патологией, р<0,001 (см. раздел «обсуждение»).

Медиана заболеваемости зобом за 10 лет составила 202,9 случая на 100 000 человек, медиана ежегодного падения заболеваемости — 6,1 случая на 100 000 человек.

В изучаемый период отмечается статистически значимый рост заболеваемости тиреоидитом, р<0,001 (рис. 7). Медиана заболеваемости за 10 лет составила 44,8 случая на 100 000 человек, медиана ежегодного прироста заболеваемости — 1,6 случая на 100 000 человек.

За десятилетний период отмечен статистически значимый рост заболеваемости гипотиреозом, р<0,001 (рис. 8). Медиана заболеваемости за 10 лет составила 50,2 случая на 100 000 человек, медиана ежегодного прироста заболеваемости — 2,9 случая на 100 000 человек.

На территории РФ в изучаемый период зафиксирован статистически значимый рост заболеваемости тиреотоксикозом, p=6,4×10-3 (рис. 9). Медиана заболеваемости за 10 лет составила 15,5 случая на 100 000 человек. Медиана ежегодного прироста заболеваемости составила 0,3 случая на 100 тыс. человек.

На рис. 10 представлена динамика заболеваемости синдромом врожденной йодной недостаточности: зафиксирована тенденция к росту, однако данные статистически не значимы, р=0,230. Медиана заболеваемости за 10 лет составила 0,4 случая на 100 000 человек, медиана ежегодного прироста заболеваемости — 0,04 случая на 100 000 человек.

**Figure:**
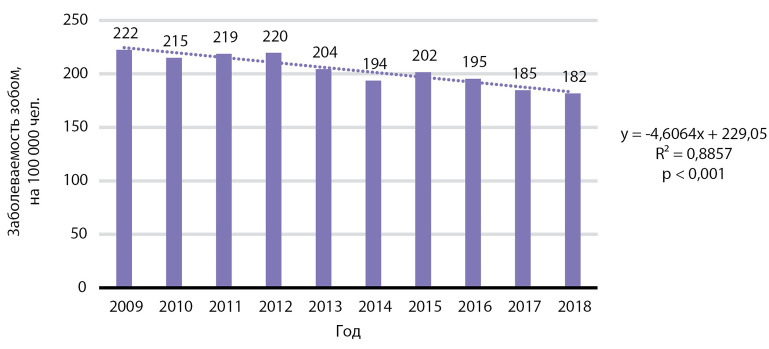
Рисунок 6. Динамика заболеваемости зобом, на 100 000 человек.

**Figure:**
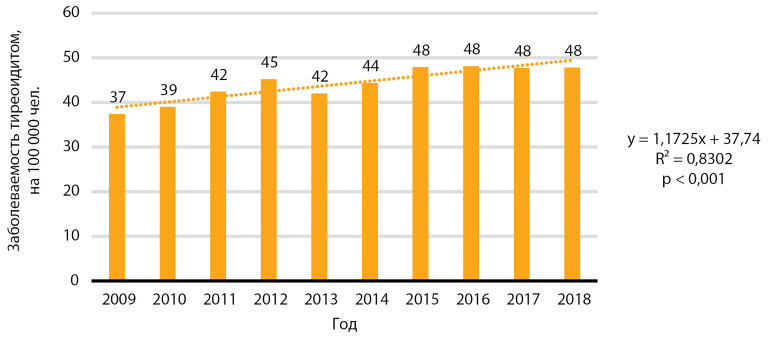
Рисунок 7. Динамика заболеваемости тиреоидитом, на 100 000 человек.

**Figure:**
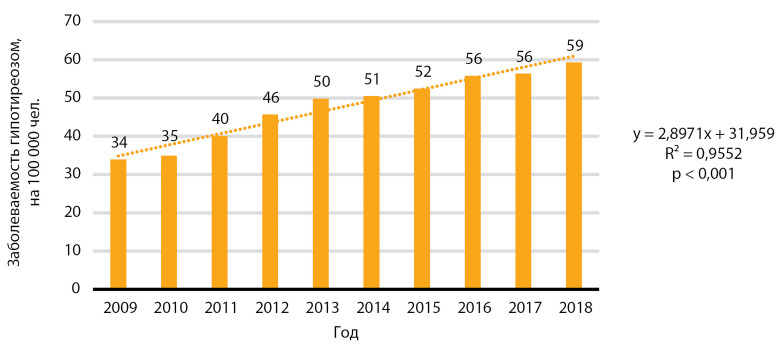
Рисунок 8. Динамика заболеваемости гипотиреозом, на 100 000 человек.

**Figure:**
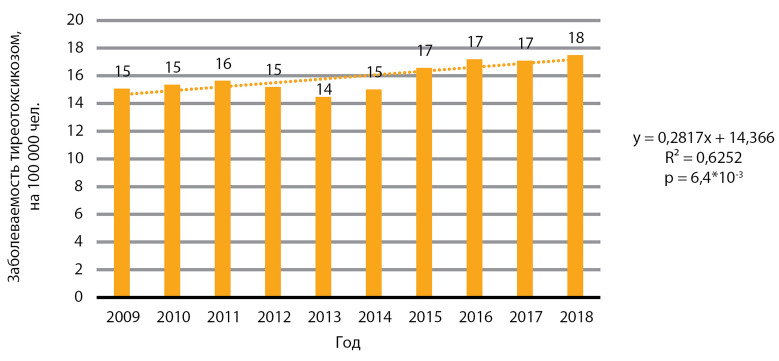
Рисунок 9. Динамика заболеваемости тиреотоксикозом, на 100 000 человек.

**Figure:**
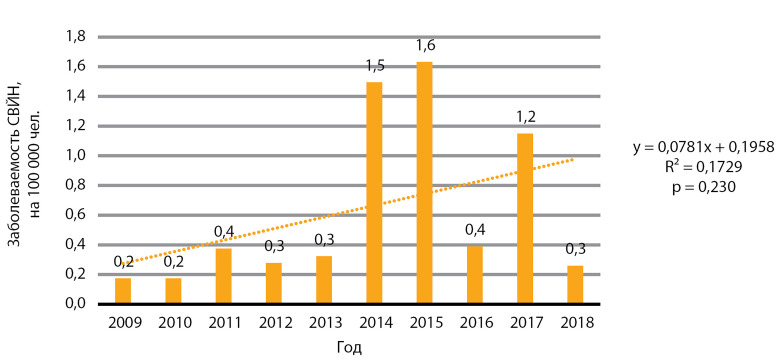
Рисунок 10. Динамика заболеваемости синдромом врожденной йодной недостаточности (СВЙН), на 100 000 человек.

## ОБСУЖДЕНИЕ

Среди возможных недостатков исследования следует отметить тот факт, что данные официальной государственной статистики базируются на сведениях, поступающих из различных регионов РФ, вероятно, имеющих различные особенности кодирования диагнозов по МКБ-10.

## Резюме основного результата исследования

Таким образом, выявлен статистически значимый рост распространенности различных форм зоба, тиреотоксикоза, синдрома йодной недостаточности, статистически значимый рост заболеваемости тиреотоксикозом. Отмечено снижение заболеваемости различными формами зоба. В отношении заболеваемости синдромом йодной недостаточности также получен рост, однако данные статистически незначимы.

## Обсуждение основного результата исследования

По мнению авторов, положительная (рост распространенности/заболеваемости) или отрицательная (снижение распространенности/заболеваемости) динамика свидетельствует о неэффективности или эффективности соответственно проводимых профилактических мероприятий по борьбе с ЙД.

Выявленный нами статистически значимый рост распространенности и заболеваемости указанных заболеваний можно рассматривать как компенсаторную реакцию на сохраняющийся дефицит йода на территории РФ. Статистически значимый рост распространенности и заболеваемости тиреоидитом можно объяснить ростом аутоиммунной патологии, совершенствованием уровня диагностики, а также, в некоторых случаях, гипердиагностикой (например, при постановке диагноза аутоиммунного тиреоидита у лиц с бессимптомным носительством антитиреоидных антител). В то же время в популяции отмечена тенденция к снижению заболеваемости зобом. При этом следует отметить, что, несмотря на намеченную тенденцию, его показатель у всего населения РФ не достиг своего спорадического уровня, что свидетельствует о неудовлетворительной организации профилактической работы по устранению ЙД в большинстве регионов РФ.

Кроме того, на примере стран, имеющих законодательное всеобщее йодирование соли, продемонстрированы значительные успехи.

Ранее авторы сообщали о снижении заболеваемости диффузным эндемическим зобом в республике Беларусь: более чем в 10 раз за 13 лет на фоне программы обязательного йодирования соли [12].

В 1994 г. программа всеобщего йодирования соли была принята в Пакистане. Позднее для оценки состояния питания (включая дефицит йода) населения были проведены два национальных исследования: первое в 2001 г., а второе — спустя десятилетие, в 2011 г. [13][14]. Результаты этих исследований выявили существенные улучшения. Распространенность йододефицита у детей школьного возраста (6–12 лет) была снижена до 36,7% в 2011 г. с 63,7% в 2001 г., а распространенность среди женщин репродуктивного возраста (15–49 лет) снизилась с 76,3 до 48%. Усилия, предпринятые в течение десятилетия, привели к снижению распространенности ЙДЗ на 50% [15], и страна получила статус адекватного йодного обеспечения, согласно рекомендуемым ВОЗ критериям.

Высокая распространенность зоба, вызванного ЙД (среднее содержание йодида калия — 5,6 мг/кг соли, медианная концентрация йода в моче составляла 68 мкг/л), наблюдалась в Хорватии по данным исследований 1991 г. и 1995 г., когда содержание йодида калия в соли составляло 10 мг/кг соли. В новом постановлении, введенном в 1996 г., содержание йодида калия в соли составило 25 мг/кг соли, что привело к увеличению медианной концентрации йода в моче до 248 мкг/л. Впоследствии, до 2018 г., распространенность зоба оценивалась только в двух небольших исследованиях. В 2018 г. проведено исследование, изучавшее распространенность и этиологию зоба у 3594 школьников через 17 лет после увеличения концентрации йодида калия в соли. Зоб был обнаружен у 32 детей (0,89% против 2,8% в 1991 г., p<0,00001, и 27% в 1995 г., p<0,00001). Субклинический гипотиреоз обнаружен у троих детей. Авторы делают вывод, что повышенное поступление йода снижает распространенность зоба [5].

Известно, что заболеваемость умеренным гипертиреозом выше в районах с ЙД, чем в районах с достаточным содержанием йода, и снижается после внедрения универсальных программ йодирования соли [16].

На территориях с адекватным потреблением йода приблизительно 80% гипертиреоза приходится на долю пациентов с болезнью Грейвса, в то время как в районах с ЙД токсический многоузловой зоб и токсическая аденома составляют 50% всех случаев гипертиреоза [17]. Это обстоятельство обусловлено тем, что на фоне длительно существующего ЙД сформировавшиеся узлы ЩЖ со временем приобретают автономию и вырабатывают тиреоидные гормоны, независимо от влияния тиреотропного гормона [18][19].

Массовую йодную профилактику на сегодняшний день успешно проводят в большинстве стран мира, в том числе в США, Канаде, Китае, европейских странах.

Распространенность зоба в материковом Китае сократилась почти вдвое после введения в 1996 г. программы всеобщего йодирования соли. Авторы отмечают, что избыток йода также может вызывать развитие заболеваний ЩЖ, в связи с чем стандарты йодирования соли должны быть установлены в соответствии с местными условиями [20].

Среди недавно опубликованных работ стоит отметить, к примеру, ситуацию на Шри-Ланке, где всеобщее йодирование соли было введено в 1995 г. При проведении массового исследования (16 910 школьников) распространенность зоба при пальпации была значительно снижена — с 18,6% до 2,1% (р<0,05) [21].

Другие страны, не имеющие законодательного регулирования проблемы ЙД, также сообщают о необходимости его введения [22].

## ЗАКЛЮЧЕНИЕ

В результате анализа динамики эпидемиологических показателей показан рост распространенности зоба, синдрома врожденной йодной недостаточности у всего населения РФ, что свидетельствует об отсутствии положительной динамики на фоне проводимых региональных мероприятий. Также продемонстрирован рост распространенности тиреоидита, тиреотоксикоза; рост заболеваемости тиреоидитом, гипотиреозом, тиреотоксикозом. Существующие на сегодняшний день подходы к йодной профилактике недостаточно эффективны, что диктует необходимость внедрения всеобщего обязательного йодирования соли на территории РФ.
